# Reliability of Calculation of Dynamic Modulus for Asphalt Mixtures Using Different Master Curve Models and Shift Factor Equations

**DOI:** 10.3390/ma15124325

**Published:** 2022-06-18

**Authors:** Hao Chen, Diego Maria Barbieri, Xuemei Zhang, Inge Hoff

**Affiliations:** 1 Department of Civil and Environmental Engineering, Norwegian University of Science and Technology, Høgskoleringen 7A, 7491 Trondheim, Norway; diegomb271@gmail.com (D.M.B.); xuemei.zhang@ntnu.no (X.Z.); inge.hoff@ntnu.no (I.H.); 2 Department of Mechanical and Structural Engineering and Materials Science, University of Stavanger, Kristine Bonnevies vei 22, 4021 Stavanger, Norway

**Keywords:** asphalt mixture, dynamic modulus, master curve model, shift factor, goodness of fit

## Abstract

To develop a mechanistic-empirical pavement design system for Norwegian conditions, this paper evaluates the influence of the adoption of different models and shifting techniques on the determination of dynamic modulus master curves of asphalt mixtures. Two asphalt mixture types commonly used in Norway, namely Asphalt Concrete (AC) and Stone Mastic Asphalt (SMA) containing neat bitumen and polymer-modified bitumen, were prepared by the roller compactor, and their dynamic moduli were determined by the cyclic indirect tensile test. The dynamic modulus master curves were constructed using the standard logistic sigmoidal model, a generalized logistic sigmoidal model and the Christensen–Anderson–Marasteanu model. The shifting techniques consisted of log-linear, quadratic polynomial function, Arrhenius, William–Landel–Ferry and Kaelble methods. The absolute error, normalised square error and goodness-of-fit statistics encompassing standard error ratio and coefficient of determination were used to appraise the models and shifting methods. The results showed that the standard logistic sigmoidal model and the Williams–Landel–Ferry equation had the most suitable fits for the specimens tested. The asphalt mixtures containing neat bitumen had a better fit than the ones containing polymer-modified bitumen. The Kaelble equation and log-linear equation led to similar results. These findings provide a relevant recommendation for the mechanistic-empirical pavement design system.

## 1. Introduction

To develop the mechanistic–empirical pavement design system for Norwegian conditions envisaged by the Norwegian Public Roads Administration (NPRA) [1], the mechanical characterization of asphalt pavement is of primary importance. The asphalt material is sensitive to the temperature and the rate-of-load due to its viscoelastic properties [2,3]. Based on the Mechanistic–Empirical Pavement Design Guide (MEPDG) [4], the dynamic modulus is a relevant parameter to characterise the mechanical properties of asphalt pavements. Due to the time-consuming process of specimen preparation and testing, master curves were developed according to the time–temperature superposition principle [5,6]. It is necessary to select an appropriate method to evaluate the dynamic modulus master curve among all the formulated extrapolation methodologies in order to develop a proper mechanistic–empirical pavement design system.

The Standard Logistic Sigmoidal (SLS) model used in the MEPDG is widely used to construct the dynamic modulus master curve of asphalt materials. The SLS model can more accurately fit the dynamic modulus test data in a wider temperature range than a single polynomial model [7,8]. However, the SLS model is more applicable when test data are symmetrical, whereas the Generalized Logistic Sigmoidal (GLS) model developed by Rowe et al. is better employed to fit asymmetric data [9,10]. Moreover, Marasteanu and Anderson proposed a Christensen–Anderson–Marasteanu (CAM) model based on the Christensen–Anderson (CA) formulation [11], which provides a better fit of the dynamic modulus of asphalt mixtures within very low and very high frequencies for unmodified and polymer modified bituminous binders [12,13,14].

To construct the dynamic modulus master curve, different shifting techniques (shift factor, *α_T_*) were used to model the time–temperature superposition relationship related to the viscoelastic properties of asphalt materials. Traditionally, the temperature dependence of relaxation modes in the vicinity of glass transition temperature (*T_g_*) is modelled with the Williams–Landel–Ferry (WLF) equation [15]. However, recent experimental studies [16,17] found that the temperature dependence of viscoelastic properties deviate from the WLF equation below *T_g_*. To address this issue, the Kaelble equation based on the WLF formulation was proposed [18,19,20]. The Kaelble equation is a symmetric function devised to reflect the temperature dependence of viscoelastic properties below *T_g_* [21]. The Arrhenius equation is another popular model used to describe the temperature dependence of viscoelastic properties of materials. The WLF and Arrhenius equations focus on the volume processes and the thermally activated processes, respectively [10,22]. In addition to the above two methods, the log-linear equation can also describe the temperature dependence of asphalt materials [7]. The log-linear equation presents the straight-line relationship between *log(α_T_)* and temperature, and is normally used for asphalt mixtures [23]. The quadratic polynomial equation is another well-known shift factor equation that can accurately fit the shift factors over a wide range of temperatures [24,25,26].

The objective of this study is to adopt various models and shift factor equations to evaluate the dynamic modulus of four types of asphalt mixtures commonly used in Norwegian highway by means of Cyclic Indirect Tensile Tests (CITTs) conducted in the laboratory. The three master curve models and five shift factor equations were employed to assess the dynamic modulus master curve. The quality of the extrapolation calculations was assessed by error analysis and goodness-of-fit statistics. The results can provide recommendations for the selection of the proper master curve model and shift factor equation for developing the mechanistic–empirical pavement design system.

## 2. Materials and Methods

### 2.1. Materials

The Neat Bitumen (NB) of Pen 70/100 and the Polymer Modified Bitumen (PMB) of Pen 65/105 were supplied by Veidekke company (Trondheim, Norway) and Nynas company (Göteborg, Sweden), respectively. Their main physical properties are given in Table 1. In PMB, the critical network structure is formed between the polymer molecule and the asphalt binder, which enhances its deformation resistance at high temperature, resulting in a lower penetration and a higher softening point [27].

Crushed rock aggregates supplied by Franzefoss company (Heimdal, Norway) were adopted, and their resistance to wear and fragmentation are specified in Table 2. The aggregates fulfilled the requirements for AC and SMA mixture with an Annual Average Daily Traffic (AADT) higher than 15,000 [30].

### 2.2. Cyclic Indirect Tensile Test

#### 2.2.1. Sample Preparation

**Figure 1:**
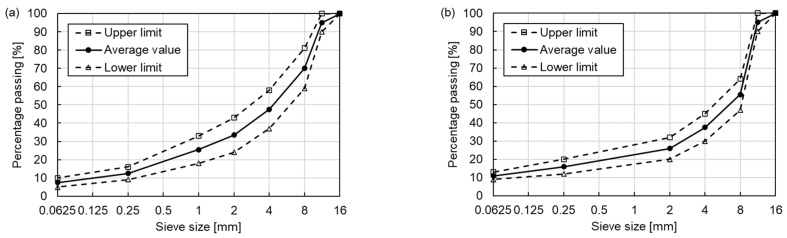
Gradation curves of (**a**) AC 11 and (**b**) SMA 11.

The asphalt mixture specimens were prepared in the laboratory based on the average value of the upper limit and lower limit in the gradation curves of AC 11 and SMA 11 shown in Figure 1 [30], and the Optimum Binder Contents (OBC, by asphalt mixture weight) were determined by the Marshall mix design. Therefore, four types of mixtures were used in this study, as shown in Table 3. The OBC of AC 11-NB, AC 11-PMB, SMA 11-NB and SMA 11-PMB were, respectively, 5.1%, 5.2%, 5.3% and 5.3%.

The asphalt slabs were compacted using a roller compactor based on the gradation curves and OBC of asphalt mixtures. Then, designated specimens with a diameter of 100 mm and a height of 40 mm were drilled, cut and further used to perform CITT. A total of 16 specimens were prepared (four replicate specimens for each type of mixture). The maximum density of each asphalt mixture was determined by its aggregate density and its bitumen density based on the mathematic procedure method. The void characteristics of specimens are given in Table 4.

#### 2.2.2. Testing Procedure

The CITT was performed using the Nottingham Asphalt Tester (NAT) produced by Cooper Technology Company (Ripley, United Kingdom). The controlled harmonic sinusoidal load was applied without rest period through a servo-controlled double acting pneumatic actuator. The horizontal deformation was detected by two Linear Variable Differential Transformers (LVDT). The sets of frequencies and temperatures were, respectively, 10 Hz, 5 Hz, 3 Hz, 1 Hz, 0.3 Hz, 0.1 Hz and −15 °C, −10 °C, 0 °C, 15 °C, 30 °C for each test. The applied load ensured that the tested samples were in the linear viscoelastic range as the initial horizontal strain was in a range between 50 με to 100 με for each temperature and frequency. This research presents the average results deriving from the testing of four replicate specimens.

### 2.3. Master Curve Construction

#### 2.3.1. Master Curve Models

##### SLS Model

**Figure 2:**
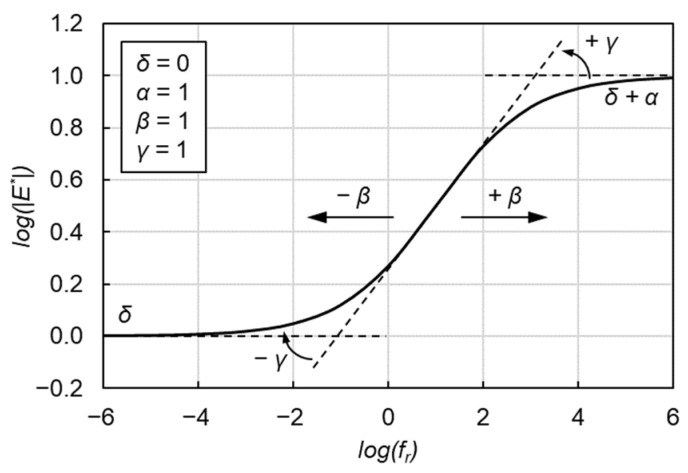
Graphical interpretation of SLS model.

The SLS model used in the MEPDG is one of the most popular models used to describe the rheological properties of asphalt mixture. The SLS model is given by Equation (1).

(1) $$\log\left(\left|E^{*}\right|\right)=\delta +\frac{\alpha }{1+e^{\beta -\gamma \cdot \log\left(f_{r}\right)}}$$

where |E*| is the dynamic modulus, *f_r_* is the frequency at the reference temperature of 15 °C in this research, and *δ*, *α*, *β* and *γ* are the fitting parameters. *δ* and *δ* + *α* represent the minimum and maximum values of |E*|, respectively. *β* and *γ* describe the shape of the SLS model as depicted in Figure 2.

##### GLS Model

**Figure 3:**
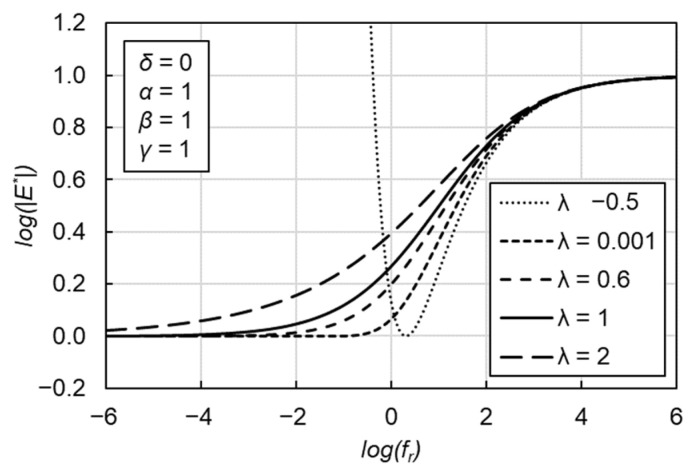
Graphical interpretation of the role of *λ* coefficient in GLS model.

The SLS model provides an excellent fit to symmetric experimental data points, but it cannot acceptably fit non-symmetric curves. Therefore, the use of a GLS model was recommended by Rowe et al., as it is the general form of sigmoidal function applicable to asymmetric curves [33] as given by Equation (2).

(2) $$\log\left(\left|E^{*}\right|\right)=\delta^{\prime }+\frac{\alpha^{\prime }}{{\left[1+\lambda \cdot e^{\beta^{\prime }-\gamma^{\prime }\cdot \log\left(f_{r}\right)}\right]}^{\frac{1}{\lambda }}}$$

where *δ*′, *α*′, *β*′, *γ*′ and *λ* are the fitting parameters. *λ* characterizes the asymmetric characteristics shown in Figure 3. *δ*′ and *δ′ + α*′ represent the minimum and maximum values of |E*|, respectively. *β*′ and *γ*′ describe the shape of the GLS model.

##### CAM Model

**Figure 4:**
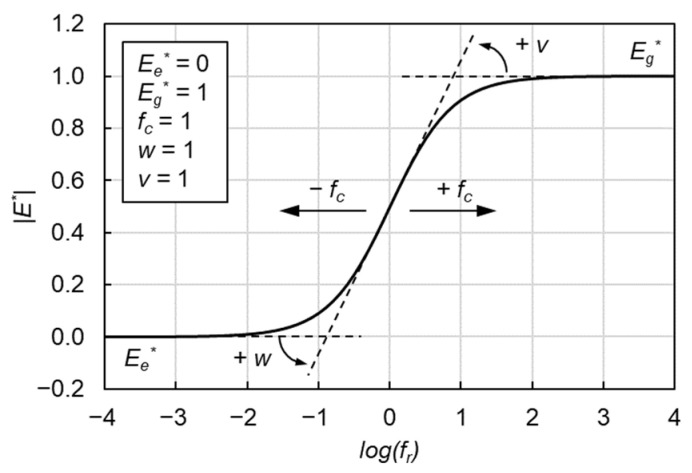
Graphical interpretation of CAM model.

The CAM model given by Equation (3) can also satisfactorily describe the viscoelastic properties of asphalt mixtures.

(3) $$\left|E^{*}\right|=E_{e}^{*}+\frac{E_{g}^{*}-E_{e}^{*}}{{\left[1+{\left(\frac{f_{c}}{f_{r}}\right)}^{v}\right]}^{w/v}}$$

where $E_{e}^{*}$ is the equilibrium modulus representing the minimum modulus, $E_{g}^{*}$ is the glassy modulus representing maximum asymptotic modulus, *f_c_* is the location parameter with dimensions of frequency, *v* and *w* are fitting parameters and describe the shape of the model as shown in Figure 4.

#### 2.3.2. Shift Factor Equations

The shift factor describes the temperature dependency of the dynamic modulus and the general form is given in Equation (4). It can be used to shift the dynamic modulus at different test temperatures to the reduced frequency of the master curve based on the reference temperature of 15 °C.

(4a) $$f_{r}=f\cdot \alpha_{T}$$

(4b) $$\log\left(f_{r}\right)=\log\left(f\right)+\log\left(\alpha_{T}\right)$$

Five commonly used shift factor equations were adopted in this research, which were the log-linear equation, quadratic polynomial equation, Arrhenius equation, WLF equation and Kaelble equation.

##### Log-Linear Equation

The log-linear equation is one of the most popular temperature-shifting methods for asphalt mixtures. Christensen and Anderson [11] suggested that below 0 °C, *log(α_T_)* varies linearly with temperature for many binders, and this same relationship has been deemed suitable for asphalt mixture at low to intermediate temperatures [34]. The log-linear equation for calculating the shift factor is:

(5) $$\log\left(\alpha_{T}\right)=C\cdot \left(T-T_{r}\right)$$

where *α_T_* is the shift factor, *T* is the temperature, *T_r_* is the reference temperature (15 °C), *C* is the constant which is determined by analysis of the experimental data.

##### Quadratic Polynomial Equation

The quadratic polynomial equation can well fit the shift factors over a wide range of temperatures, and is expressed as:

(6) $$\log\left(\alpha_{T}\right)=a\cdot \left(T-T_{r}\right)+b\cdot {\left(T-T_{r}\right)}^{2}$$

where *a* and *b* are regression parameters.

##### Arrhenius Equation

The Arrhenius equation for calculating the shift factor is presented in Equation (7):

(7) $$\log\left(\alpha_{T}\right)=C^{\prime }\cdot \left(\frac{1}{T}-\frac{1}{T_{r}}\right)=\frac{0.4347\cdot E_{a}}{R}\cdot \left(\frac{1}{T}-\frac{1}{T_{r}}\right)$$

where *C*′ is a constant, *E_a_* is the activation energy (J/mol) and *R* is the ideal gas constant (8.314 J/mol·K). The Arrhenius equation has only one constant to be determined and can describe the behaviour of the material below *T_g_* [16].

##### WLF Equation

The WLF equation is widely used to describe the relationship between shift factor and temperature above *T_g_* and thereby assess the shift factor of asphalt mixtures:

(8) $$\log\left(\alpha_{T}\right)=\frac{-C_{1}\cdot \left(T-T_{r}\right)}{C_{2}+\left(T-T_{r}\right)}$$

where *C*_1_ and *C*_2_ are two regression parameters.

##### Kaelble Equation

The Kaelble equation is a modification of the WLF equation and can describe the relationship between shift factor and temperature below *T_g_* as given in Equation (9).

(9) $$\log\left(\alpha_{T}\right)=\frac{-C_{1}{}^{\prime }\cdot \left(T-T_{r}\right)}{C_{2}{}^{\prime }+\left|T-T_{r}\right|}$$

where $C_{1}{}^{\prime }$ and $C_{2}{}^{\prime }$ are two regression parameters.

#### 2.3.3. Fitting Procedure

To construct the master curves deriving from the experimental data, the nonlinear least squares regression analysis was integrated in the Microsoft Excel Solver tool. The Sum of Square Error (*SSE*) between measured values after shifting, |E*|_measured_, and predicted values, |E*|_predicted_, as shown in Equation (10) was used for the fitting procedure.

(10) $$SSE=\sum \frac{{\left({\left|E^{*}\right|}_{measured}-{\left|E^{*}\right|}_{predicted}\right)}^{2}}{{\left({\left|E^{*}\right|}_{measured}\right)}^{2}}$$

To define the optimal results of master curves, the coefficients of the models and shift factor equations were fitted to minimize *SSE*. The constraint range of variables was not defined due to well fitting results for the cases. The selection of solving method was GRG Nonlinear. Furthermore, the same initial values of fitting parameters were used for each fitting procedure.

### 2.4. Goodness of Fit Statistics

The standard error ratio and coefficient of determination (*R*^2^) were used to evaluate the goodness of fit between measured and predicted values. The standard error of estimation and standard error of deviation are defined as follows [35]:

(11) $$S_{e}=\sqrt{\frac{{\sum \left(Y-\hat{Y}\right)}^{2}}{\left(n-k\right)}}$$

(12) $$S_{y}=\sqrt{\frac{{\sum \left(Y-\bar{Y}\right)}^{2}}{\left(n-1\right)}}$$

where *S_e_* is the standard error of estimation, *S_y_* is the standard error of deviation, *n* is sample size, *k* is the number of independent variables, *Y* is the measured value, *Ŷ* is the predicted value and *Ȳ* is the average value of measured values. The standard error ratio is defined as *S_e_/S_y_*. *R*^2^ is determined as follows:

(13) $$R^{2}=1-\frac{\left(n-k\right)}{\left(n-1\right)}\cdot {\left(\frac{S_{e}}{S_{y}}\right)}^{2}$$

Lower *S_e_/S_y_* and higher *R*^2^ values indicate better goodness between predicted and measured data. Based on the criteria of the goodness of fit from previous research [36], *S_e_/S_y_* and *R*^2^ of this research are lower than 0.35 and higher than 0.90, respectively, which indicates that all results have a good fit.

## 3. Results and Discussion

### 3.1. Dynamic Modulus Master Curve

**Figure 5:**
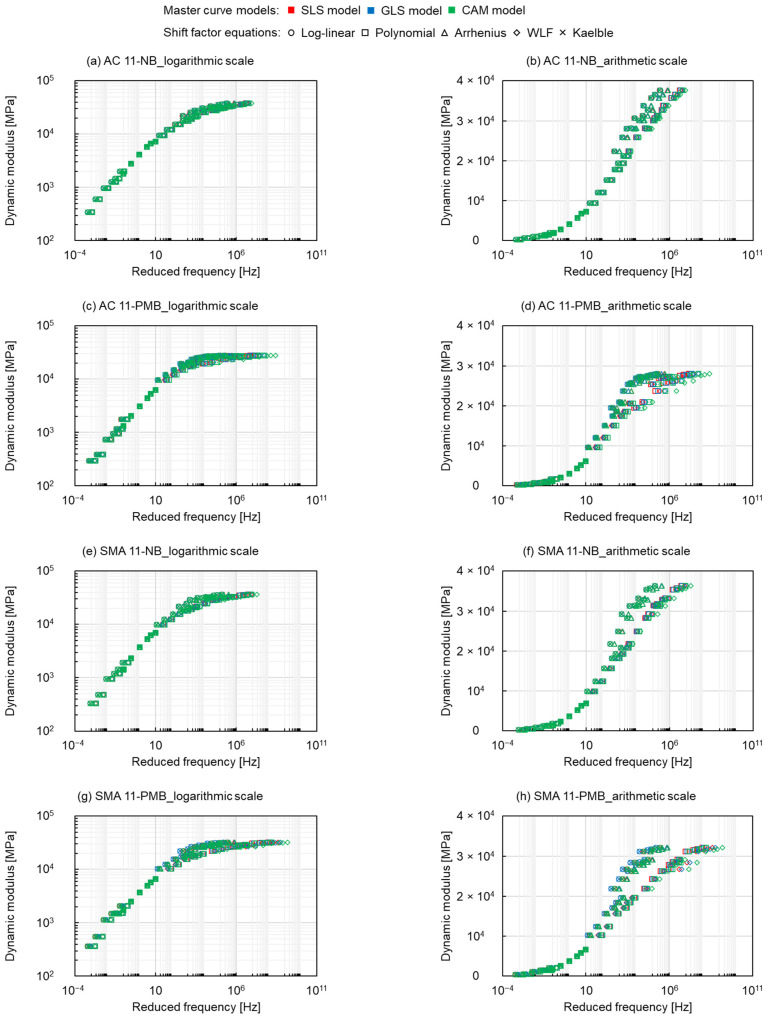
Dynamic modulus master curves of (**a**) and (**b**) AC 11-NB, (**c**) and (**d**) AC 11-PMB, (**e**) and (**f**) SMA 11-NB and (**g**) and (**h**) SMA 11-PMB.

The fitting results of master curves are presented in Figure 5, where red represents the SLS model, blue represents the GLS model and green represents the CAM model, moreover, circle markers, square markers, triangle markers, diamond markers and crosses represent the log-linear equation, the polynomial equation, the Arrhenius equation, the WLF equation and the Kaelble equation, respectively. The results of the three model fits are similar for the four types of asphalt mixtures. The differences appear in the various shift factor equations. No matter at high or low reduced frequency, the dynamic modulus values fitted by the log-linear equation, Arrhenius equation and Kaelble equation are bigger than the ones fitted by the polynomial equation and WLF equation. The fitting parameters of the log-linear equation and the Kaelble equation are similar, and the fitting parameters of the polynomial function and the WLF equation are very close.

**Figure 6:**
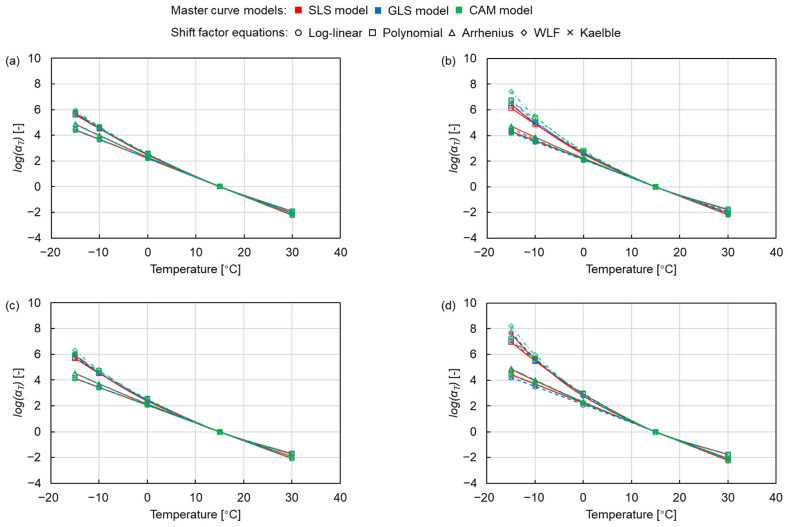
Shift factors of (**a**) AC 11-NB, (**b**) AC 11-PMB, (**c**) SMA 11-NB and (**d**) SMA 11-PMB.

Figure 6 shows the changes in shift factors with temperature, where the colour and shape of the marker represent the same master curve models and shift factor equations as in Figure 5. The curves of the log-linear equation, Arrhenius equation and Kaelble equation are relatively close and show a linear shape, while the curves of the polynomial equation and the WLF equation are similar and more curved than the former ones. This indicates that the polynomial equation and the WLF equation shift the curve more to the right at high reduced frequency and more to the left at low reduced frequency, as shown in Figure 5, resulting in the higher dynamic modulus values fitted by the log-linear equation, the Arrhenius equation and the Kaelble equation. Meanwhile, the difference in the shift factor of the PMB asphalt mixtures is more obvious than the one of the NB asphalt mixtures, and the SMA mixtures have a more pronounced effect on the difference between shift factors than the AC mixtures. This indicates that the PMB and the SMA mixtures are more sensitive to the shift factor equations. This can be explained considering that the different cross-linked structures of the NB and the PMB and the different skeleton structures of the SMA and the AC mixtures lead to the distinction in the mechanical response of the asphalt mixtures, which causes the difference in the shift factor equations on the modelling of dynamic modulus.

**Figure 7:**
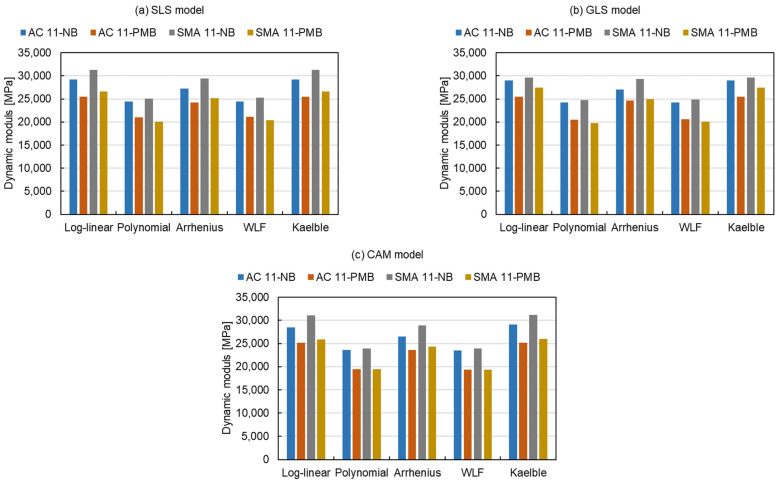
Modelling values of dynamic modulus at the reduced frequency of 10^4^ Hz (*T_r_* = 15 °C).

Figure 7 presents the modelling values of the dynamic modulus at the reduced frequency of 10^4^ Hz (*T_r_* = 15 °C) for three master curve models and five shift factor equations. The distinction of dynamic modulus is not greatly affected by the master curve models. The influence of the five shift factor equations can be divided into two categories. The first category including the log-linear equation, Arrhenius equation and Kaelble equation, shows a higher value in dynamic modulus. Another category including the polynomial equation and the WLF equation exhibits a lower dynamic modulus of mixtures. The dynamic modulus of the former category is on average about 23% higher than that of the latter one.

**Figure 8:**
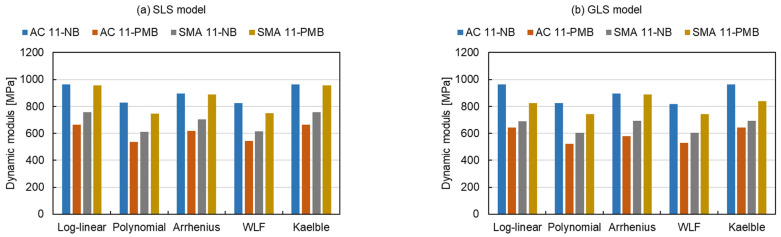

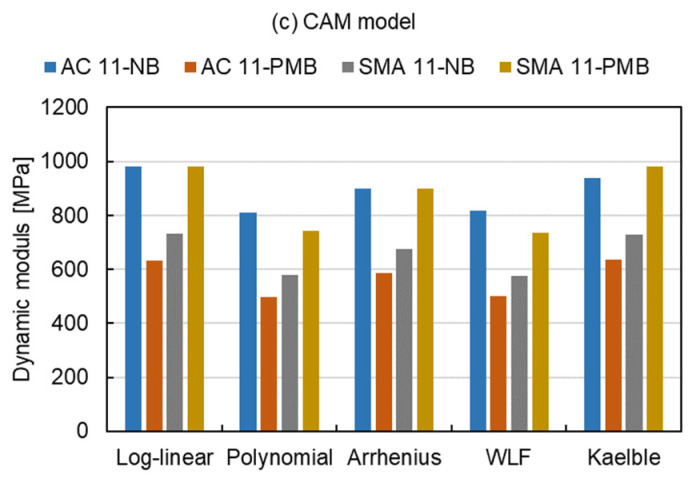
Modelling values of dynamic modulus at the reduced frequency of 10^−2^ Hz (*T_r_* = 15 °C).

The modelling values of the dynamic modulus at the reduced frequency of 10^−2^ Hz (*T_r_* = 15 °C) are given in Figure 8 and have a similar trend as the results at 10^4^ Hz, which can also be divided into the same two categories. The former one has an average 19% higher dynamic modulus than the latter one. The results of Figure 7 and Figure 8 indicate that the modelling values of dynamic modulus fitted by the log-linear equation, Arrhenius equation and Kaelble equation are approximately 20% higher than the ones fitted by the polynomial equation and the WLF equation, both at high and low reduced frequencies.

### 3.2. Error Analysis

#### 3.2.1. Absolute Error

**Figure 9:**
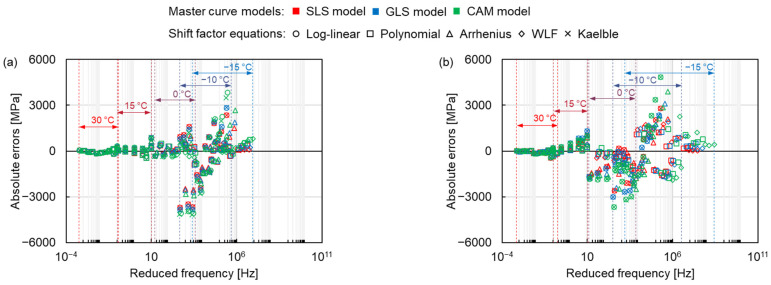

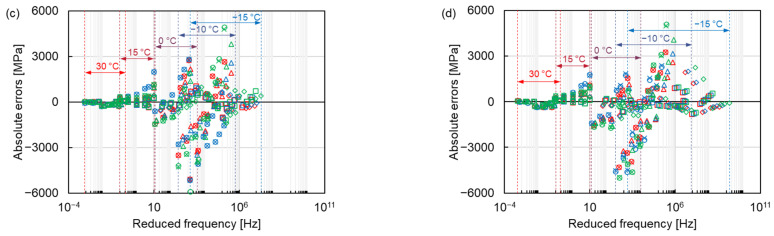
Absolute errors of (**a**) AC 11-NB, (**b**) AC 11-PMB, (**c**) SMA 11-NB and (**d**) SMA 11-PMB.

After comparing the master curves constructed by different models, the error analysis of each model and shift factor equation was carried out. The absolute errors of the dynamic modulus between the modelling values fitted by the three master curve models and the five shift factor equations, and the measured values are shown in Figure 9, where the colour and shape of the marker represent the same master curve models and shift factor equations as in Figure 5. The absolute error is small at high temperatures and relatively big at the temperature range between −15 °C to 0 °C for all mixtures. The maximum absolute error at −10 °C can be explained by the connection between viscoelastic stage and elastic stage, which in turn changes the mechanical response of mixtures. As the temperature continues to decrease (the reduced frequency increases), the absolute error becomes smaller again at −15 °C (higher reduced frequency). This result is attributed to the elastomer of asphalt mixture at very low temperature, resulting in a constant dynamic modulus.

**Figure 10:**
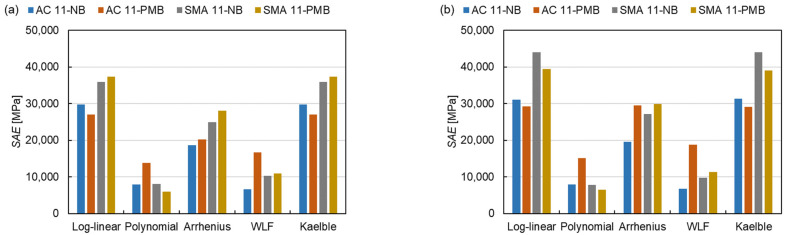

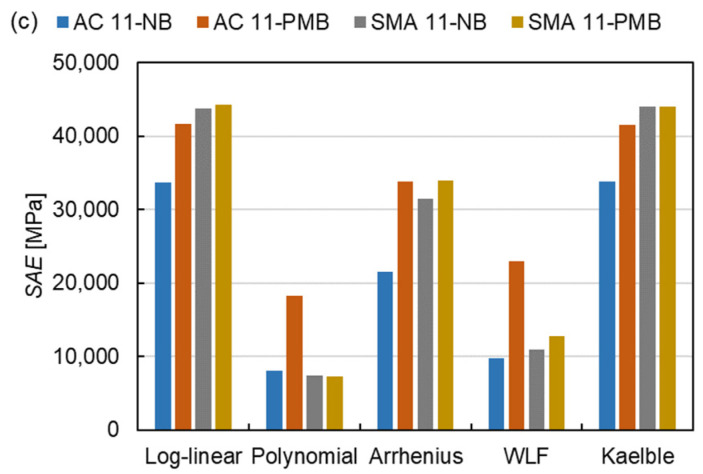
*SAE* of all fitting procedures: (**a**) SLS model, (**b**) GLS model and (**c**) CAM model.

The shift factor equation has more influence on the Sum of Absolute Error (*SAE*) than the master curve models, as shown in Figure 10. There are 15 master curve model-shift factor equation combinations for 4 types of mixtures resulting in a total of 60 fitting procedures. The average *SAE* of the fitting procedures with the controlled fitting condition are used for comparing the distinctions of the master curve models, shift factor equations and asphalt mixture types as expressed in Equation (14).

(14) $$\bar{SAE}=\frac{\sum SAE\left(\mathrm{master}\;\mathrm{curve}\;\mathrm{models},\;\mathrm{shift}\;\mathrm{factor}\;\mathrm{equations},\;\mathrm{asphalt}\;\mathrm{mixture}\;\mathrm{types}\right)}{n}$$

where $\bar{SAE}$ is the average *SAE* and *n* is the number of fitting procedures. The $\bar{SAE}$ of the fitting procedures with the SLS model, the GLS model and the CAM model are 21,598 MPa, 23,851 MPa and 27,244 MPa, respectively. This indicates that the SLS model has the smallest absolute error, the CAM model has the largest absolute error and the GLS model is in between. Regarding the shift factor equations, the *SAE* for the log-linear equation and the Kaelble equation are similar and classified as class 1, the *SAE* for the polynomial equation and the WLF equation are close and grouped into class 2 and the value for the Arrhenius equation lies between them as class 3. The absolute error of class 1 is more than twice that of class 2. The results reveal that the SLS model and the polynomial function have the smallest absolute errors in three master curve models and five shift factor equations, respectively. For class 1, the absolute error of SMA mixtures is larger than the one of AC mixtures. Comparing four types of mixtures, the AC 11-PMB has the highest absolute error of class 2.

#### 3.2.2. Normalised Square Error

**Figure 11:**
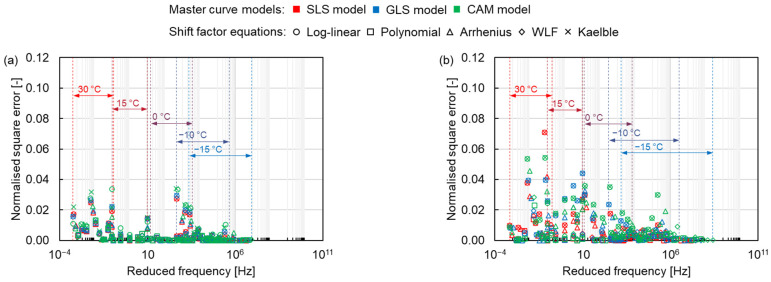

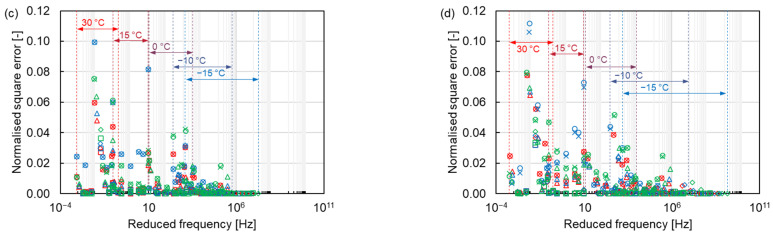
Normalised square errors of (**a**) AC 11-NB, (**b**) AC 11-PMB, (**c**) SMA 11-NB and (**d**) SMA 11-PMB.

Since the dynamic modulus of asphalt mixtures is distinct at different temperatures and frequencies, it is difficult to compare the error under the same condition over the full frequency range. The normalised square error was analysed to compare different models and shift factor equations at the same condition. From Figure 11, the normalised square error is larger at high temperatures and smaller at low temperatures, contrary to the results of the absolute error, which reflects the error of dynamic modulus at high temperatures. The maximum normalised error appears at the high temperature of 30 °C. As the temperature increases, the asphalt transitions to a viscous flow state, and its dynamic modulus is more obviously affected by the loading conditions, becoming unstable, resulting in an increasing normalised square error. Furthermore, the distinction between different asphalt mixtures can also be found. The normalised square error for asphalt mixtures containing PMB is relatively higher than the one for asphalt mixtures containing NB. Compared to the NB, the polymer molecular in the PMB also provides a portion of the stiffness modulus for the asphalt mixture. The complex connection between the polymer molecular and the asphalt binder, such as the composition and distribution of the polymer molecular in the asphalt binder, determines the stiffness modulus of the asphalt mixture [37,38]. Therefore, the dynamic modulus change of the asphalt mixture containing PMB is more complicated than that of the NB asphalt mixture, resulting in a larger error. Otherwise, the SMA mixtures have a higher normalised square error than the AC mixtures. The SMA mixture contains more coarse aggregates than the AC mixture, leading to more particle angularity. The greater the particle angularity, the higher the stiffness modulus of the asphalt mixture [39]. Thus, the change of the dynamic modulus of the SMA mixture is more complex than that of the AC mixture, resulting in a larger error.

**Figure 12:**
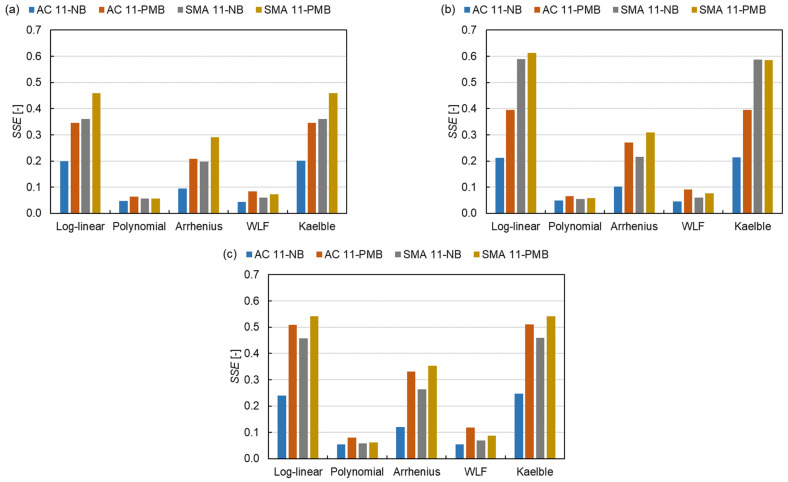
*SSE* of all fitting procedures: (**a**) SLS model, (**b**) GLS model and (**c**) CAM model.

The *SSE* for different models has the same trend as the *SAE* as shown in Figure 12. The same approach as *SAE* is used for *SSE* as shown in Equation (15).

(15) $$\bar{SSE}=\frac{\sum SSE\left(\mathrm{master}\;\mathrm{curve}\;\mathrm{models},\;\mathrm{shift}\;\mathrm{factor}\;\mathrm{equations},\;\mathrm{asphalt}\;\mathrm{mixture}\;\mathrm{types}\right)}{n}$$

where $\bar{SSE}$ is the average *SSE*. The $\bar{SSE}$ of the SLS model is 0.20, which is also smaller than those of the GLS model (0.25) and the CAM model (0.26). Based on *SSE* values, five shift factor equations are divided into three classes, the same as the classification in Section 3.2.1. The $\bar{SSE}$ of class 1 is around five times that of class 2. The results show that the SLS model and the polynomial function have the smallest normalised square errors in the three master curve models and the five shift factor equations, respectively. The mixtures containing PMB have a lower *SSE* than the mixtures containing NB, which indicates that the fit of the model for NB is better than the one for PMB, which can be explained by the effect of the PMB structure on the dynamic modulus of the asphalt mixture.

### 3.3. Goodness of Fit

**Figure 13:**
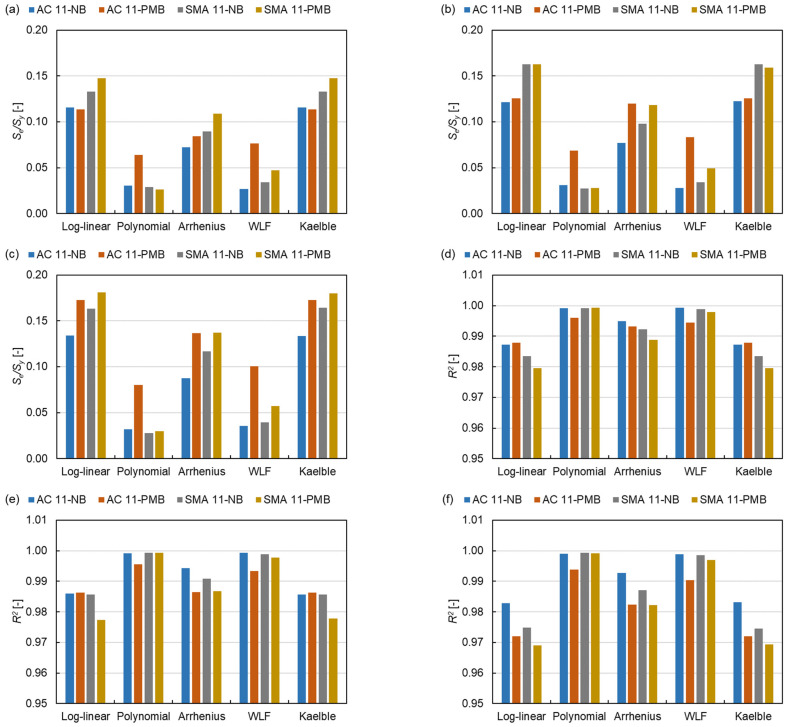
*S_e_/S_y_* of (**a**) SLS model, (**b**) GLS model and (**c**) CAM model and *R*^2^ of (**d**) SLS model, (**e**) GLS model and (**f**) CAM model.

The *S_e_/S_y_* and *R*^2^ are used to evaluate the quality of the model. From Figure 13, the SLS model has the smallest average value of *S_e_/S_y_* (0.0853) and the highest average value of *R*^2^ (0.9915) compared to the GLS model (0.0951, 0.9906) and the CAM model (0.1090, 0.9859). Otherwise, the average values of *S_e_/S_y_* and *R*^2^ for the polynomial equation are the smallest (0.0394) and the largest (0.9982), respectively. While the Kaelble equation has the biggest average value of *S_e_/S_y_* (0.1441) and the smallest average value of *R*^2^ (0.9810). These results indicate that the SLS model and the polynomial function have the best goodness-of-fit in the three master curve models and the five shift factor equations, respectively. Among the 15 kinds of fits, the NB asphalt mixture shows an overall better goodness-of-fit than the PMB asphalt mixture. This indicates that the dynamic modulus of PMB asphalt mixture is affected by more factors than that of NB asphalt mixture due to the effect of polymer molecular in the binder.

#### 3.3.1. Master Curve Models

**Figure 14:**
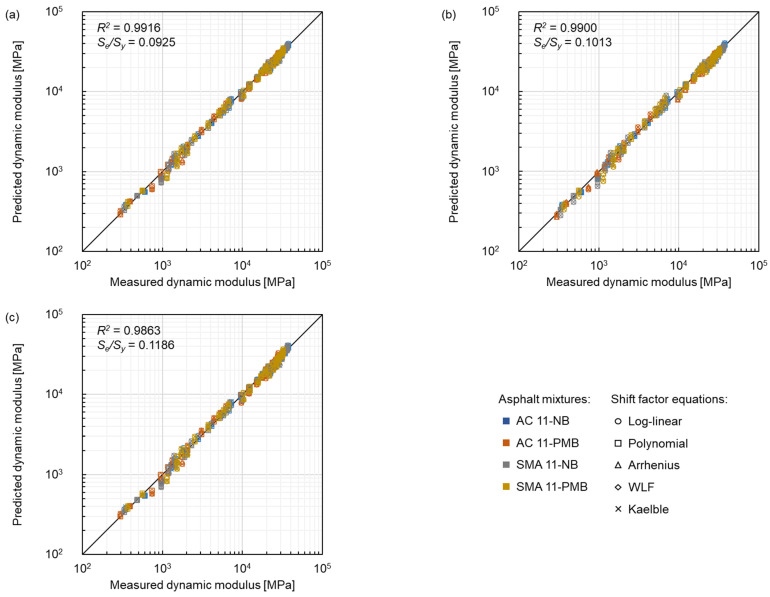
Comparison between measured dynamic modulus and predicted dynamic modulus of three master curve models: (**a**) SLS model, (**b**) GLS model and (**c**) CAM model.

The comparison of master curve models between measured dynamic modulus and predicted dynamic modulus is shown in Figure 14, where blue represents AC 11-NB, orange represents AC 11-PMB, gray represents SMA 11-NB and yellow represents SMA 11-PMB, and the shape of the marker represents the same shift factor equation as in Figure 5. All the models fit the data satisfactorily according to the goodness-of-fit ranking criteria. The SLS model had the lowest *S_e_/S_y_* of 0.0925 and the highest *R*^2^ of 0.9916, which indicates that this model shows a better goodness-of-fit than the GLS model and the CAM model under the test conditions of this study. The *S_e_/S_y_* and *R*^2^ of the CAM model were, respectively, 28.2% higher and 0.5% lower than the respective parameters of the SLS model, showing the worst correlation in the three models. Therefore, the SLS model with better goodness-of-fit can be considered for modelling the four asphalt mixtures.

#### 3.3.2. Shift Factor Equations

**Figure 15:**
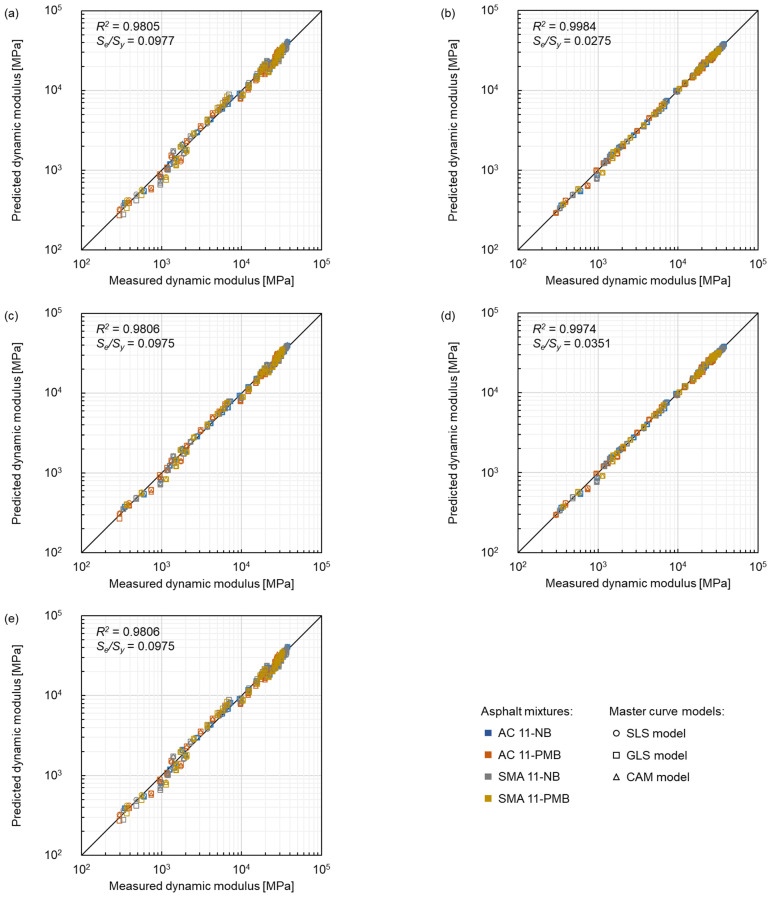
Comparison between measured dynamic modulus and predicted dynamic modulus of five shift factor equations: (**a**) log-linear equation, (**b**) quadratic polynomial equation, (**c**) Arrhenius equation, (**d**) WLF equation and (**e**) Kaelble equation.

The comparison between measured dynamic modulus and predicted dynamic modulus related to the selection of shift factor equation is shown in Figure 15, where the colour represents the same type of asphalt mixtures as in Figure 14. Furthermore, circle markers, square markers and triangle markers represent the SLS model, GLS model and CAM model, respectively. The fitting results showed that all the considered five equations had fit the data satisfactorily according to the goodness-of-fit ranking criteria. The *log(α_T_)* of the Kaelble equation showed a linear trend within the test temperature range, the fitting results of the Kaelble equation and the log-linear equation were similar, and the same findings were also shown in the former sections. The quadratic polynomial equation displayed the best goodness-of-fit with the lowest *S_e_/S_y_* of 0.0275 and the highest *R*^2^ of 0.9984. The fitting results of the WLF equation and the quadratic polynomial equation were similar and showed a good fit. The fit related to the Arrhenius equation was in the middle among the five equations. Furthermore, the transform between frequency and temperature was more convenient for the WLF equation than the quadratic polynomial due to the quadratic form. Therefore, the WLF equation was recommended for modelling the dynamic modulus of the asphalt mixtures.

### 3.4. Comparison of Fits

In this study, four indicators of absolute error, normalised square error, *S_e_/S_y_* and *R*^2^ were used to evaluate the fitting quality of the models. The 15 permutations of the three master curve models and five shift factor equations for the four types of asphalt mixtures were ranked from good to poor (from 1 to 15), and the index of fitting quality is expressed by Equation (16).

(16) $$\begin{array}{l} I_{fq}=\frac{\bar{A_{ae}}+\bar{A_{nse}}+\bar{A_{S_{e}/S_{y}}}+\bar{A_{R^{2}}}}{4}\\ \bar{A_{ae}},\;\bar{A_{nse}},\;\bar{A_{S_{e}/S_{y}}}\;and\bar{\;A_{R^{2}}}=\frac{A_{AC11-NB}+A_{AC11-PMB}+A_{SMA11-NB}+A_{SMA11-PMB}}{4}\\ A_{AC11-NB},\;A_{AC11-PMB},\;A_{SMA11-NB}\;and\;A_{SMA11-PMB}=1,\;2,\;\ldots ,\;15 \end{array}$$

where *I_fq_* is the index of fitting quality, *Ā_ae_*, *Ā_nse_*, *Ā_Se/Sy_* and *Ā*_*R*2_ are the average arrays of absolute error, normalised square error, *S_e_/S_y_* and *R*^2^, respectively, *A*_*AC* 11-*NB*_, *A*_*AC* 11-*PMB*_, *A*_*SMA* 11-*NB*_ and *A*_*SMA* 11-*PMB*_ are the arrays of the order in 15 permutations. The average values of sequences are summarised in Table 5. The shift factor equation has a more significant effect on the dynamic modulus modelling than the master curve model. The modelling of the SLS model with the polynomial equation has the best fitting quality, while the result fitted by the CAM model with the Kaelble equation is the worst in this study. Among the five shift factor equations, the polynomial equation has the best fit, followed by the WLF equation. The intertransform between frequency and temperature is more convenient for the WLF equation than the quadratic polynomial due to the quadratic form. Therefore, the SLS model and the WLF equation were recommended for modelling the dynamic modulus of these mixture types.

The same method was used to compare the models and how well the models fit different asphalt mixtures. The degree-of-fit for the four types of asphalt mixtures was ranked from good to poor (from 1 to 4) as shown in Table 6. The results indicate that the models have a better fit for asphalt mixtures containing NB than the ones containing PMB. The fitting results of AC mixtures are better than those of SMA mixtures when the bitumen is the same. These results are caused by more impact factors of the PMB asphalt mixture and the SMA mixture on the dynamic modulus.

## 4. Conclusions

In this study, the reliability of calculation of dynamic modulus using the three master curve models and the five shift factor equations was evaluated by the absolute error, normalised error and the goodness-of-fit encompassing *S_e_/S_y_* and *R*^2^ for four types of asphalt mixtures. The conclusions are summarised as follows.

- The selected shift factor equations were more influent with respect to the employed models in determining the final fitting reliability.
- The relationship between *log(α_T_)* and temperature of both the log-linear equation and the Kaelble equation were linear in the testing temperature range. These two shift factor equations had similar goodness-of-fit when extrapolating dynamic modulus master curves.
- Considering the results of absolute error, normalised square error, *S_e_/S_y_* and *R*^2^, the combination of the SLS model and the polynomial equation had the best fitting quality index (1.94), while the combination of the CAM model and the Kaelble equation had the worst fitting quality index (14.25). Regarding the different asphalt mixtures, the fitting quality index of AC 11-NB (1.45) was the best, whereas the one of SMA 11-PMB (3.25) was the worst.
- The SLS model showed the best fitting quality and was considered to model the dynamic modulus of the asphalt mixtures most used as surface layer for Norwegian highway within the investigated CITT temperature range.
- Due to better goodness-of-fit and more convenience for temperature and frequency transform, the WLF equation was considered for modelling the dynamic modulus of the asphalt mixtures most adopted in Norway.
- The master curves constructed according to all the models and all shifting techniques were characterized by better goodness-of-fit for the asphalt mixtures containing NB than the ones comprised of PMB due to the effect of PMB structure on the dynamic modulus of the asphalt mixture. The modelling of dynamic modulus master curves for SMA mixtures has a better fit than the one for SMA mixtures because of the influence of more particle angularity on the dynamic modulus of the asphalt mixture. Therefore, the models can be developed further to be suitable for the asphalt mixtures containing the PMB and SMA types of asphalt mixtures.

## Figures and Tables

**Table 1:** Physical properties of neat bitumen and PMB.

Physical Properties	Bitumen		Test Standard
	NB	PMB	
Penetration at 25 °C [0.1 mm]	92	86	EN 1426:2015 [28]
Softening point (Ring and Ball) [°C]	46.0	62.6	EN 1427:2015 [29]

**Table 2:** Resistance to wear and fragmentation of crushed rock aggregates.

Test	Value	Requirements for AADT > 15,000	Test Standard
Micro-Deval coefficient	14.2	≤20	EN 1097-1:2011 [31]
Los Angeles value	18.2	≤15	EN 1097-2:2020 [32]

**Table 3:** Designation of the tested specimens according to selected mixture types and bitumen types.

Mixture Type	Bitumen Type	Designation
AC 11	NB	AC 11-NB
	PMB	AC 11-PMB
SMA 11	NB	SMA 11-NB
	PMB	SMA 11-PMB

**Table 4:** Maximum density and void characteristics of specimens.

Mixture	Maximum Density [Mg/m^3^]	Air Voids Content [%]		Voids in Mineral Aggregate [%]		Voids Filled with Bitumen [%]	
		Value	Standard Deviation	Value	Standard Deviation	Value	Standard Deviation
AC 11-NB	2.753	3.5	0.197	16.9	0.170	79.1	0.947
AC 11-PMB	2.748	2.9	0.314	16.6	0.269	82.8	1.636
SMA 11-NB	2.740	4.4	0.252	18.1	0.216	75.8	1.101
SMA 11-PMB	2.740	3.1	0.296	17.1	0.254	81.6	1.438

**Table 5:** Comparison of fits for three master curve models and five shift factor equations.

Shift Factor Equation	Master Curve Model		
	SLS Model	GLS Model	CAM Model
Log-linear	9.75	12.56	14.00
Polynomial	1.94	2.31	3.44
Arrhenius	6.81	8.50	9.75
WLF	3.31	4.00	6.19
Kaelble	10.75	12.44	14.25

Colour bar of fitting quality: Good (1) 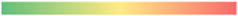 Poor (15).

**Table 6:** Comparison of fits for different asphalt mixtures.

Mixture Type	Bitumen Type	
	NB	PMB
AC 11	1.45	2.92
SMA 11	2.38	3.25

Colour bar of fitting quality: Good (1) 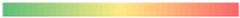 Poor (4).

## Data Availability

Not applicable.

## References

[1] NPRA VegDim. https://www.vegvesen.no/fag/fokusomrader/forskning-innovasjon-og-utvikling/pagaende-programmer-og-prosjekter/vegdim/.

[2] Bahia H.U., Zhai H., Onnetti K., Kose S. (1999). Non-linear viscoelastic and fatigue properties of asphalt binders. J. Assoc. Asph. Paving Technol..

[3] Lesueur D., Gerard J.F., Claudy P., Letoffe J.M., Planche J.P., Martin D. (1996). A structure-related model to describe asphalt linear viscoelasticity. J. Rheol..

[4] NCHRP (2004). Guide for Mechanistic-Empirical Design of New and Rehabilitated Pavement Structures.

[5] Schwartz C.W., Gibson N., Schapery R.A. (2002). Time-temperature superposition for asphalt concrete at large compressive strains. Transp. Res. Rec..

[6] Chehab G., Kim Y., Schapery R., Witczak M., Bonaquist R. (2002). Time-temperature superposition principle for asphalt concrete with growing damage in tension state. J. Assoc. Asph. Paving Technol..

[7] Pellinen T.K., Witczak M.W., Bonaquist R.F. (2004). Asphalt mix master curve construction using sigmoidal fitting function with non-linear least squares optimization. Recent Advances in Materials Characterization and Modeling of Pavement Systems.

[8] Pellinen T.K., Witczak M.W., Marasteanu M., Chehab G., Alavi S., Dongre R. (2002). Stress Dependent Master Curve Construction for Dynamic (Complex) Modulus.

[9] Rowe G., Baumgardner G., Sharrock M. (2009). Functional forms for master curve analysis of bituminous materials. Advanced Testing and Characterization of Bituminous Materials, Two Volume Set.

[10] Zhang F., Wang L., Li C., Xing Y. (2020). Predict the Phase Angle Master Curve and Study the Viscoelastic Properties of Warm Mix Crumb Rubber-Modified Asphalt Mixture. Materials.

[11] Christensen D.W., Anderson D.A. (1992). Interpretation of dynamic mechanical test data for paving grade asphalt cements (with discussion). J. Assoc. Asph. Paving Technol..

[12] Marasteanu M., Anderson D. (1999). Improved Model for Bitumen Rheological Characterization.

[13] Wathne P. (2020). Evaluation of Different Models for Construction of Master Curves for Dynamic E-module for Asphalt Materials.

[14] CholewiŃSka M., IwaŃSki M., Mazurek G. (2018). The Impact of Ageing on the Bitumen Stiffness Modulus Using the Cam Model. Balt. J. Road Bridge Eng..

[15] Williams M.L., Landel R.F., Ferry J.D. (1955). The temperature dependence of relaxation mechanisms in amorphous polymers and other glass-forming liquids. J. Am. Chem. Soc..

[16] Hutcheson S., McKenna G. (2008). The measurement of mechanical properties of glycerol, m-toluidine, and sucrose benzoate under consideration of corrected rheometer compliance: An in-depth study and review. J. Chem. Phys..

[17] O’Connell P.A., McKenna G.B. (1999). Arrhenius-type temperature dependence of the segmental relaxation below T g. J. Chem. Phys..

[18] Kaelble D.H. (1985). Computer Aided Design of Polymers and Composites.

[19] Rowe G.M., Sharrock M.J. (2011). Alternate Shift Factor Relationship for Describing Temperature Dependency of Viscoelastic Behavior of Asphalt Materials. Transp. Res. Rec. J. Transp. Res. Board.

[20] Laukkanen O.-V., Winter H.H. (2018). The dynamic fragility and apparent activation energy of bitumens as expressed by a modified Kaelble equation. J. Non-Cryst. Solids.

[21] Forough S.A., Nejad F.M., Khodaii A. (2014). A comparative study of temperature shifting techniques for construction of relaxation modulus master curve of asphalt mixes. Constr. Build. Mater..

[22] Bohn M.A. (2019). The Connection Between the Parameters of WLF Equation and of Arrhenius Equation. Propellants Explos. Pyrotech..

[23] Garcia G., Thompson M. (2007). HMA Dynamic Modulus Predictive Models (A Review).

[24] Angelone S., Borghi M., Casaux M.C., Martinez F. Evaluation of Different Procedures and Models for the Construction of Dynamic Modulus Master Curves of Asphalt Mixtures. Proceedings of the International Conferences on the Bearing Capacity of Roads Railways and Airfields.

[25] Tavassoti-Kheiry P., Boz I., Chen X., Solaimanian M. Application of ultrasonic pulse velocity testing of asphalt concrete mixtures to improve the prediction accuracy of dynamic modulus master curve. Proceedings of the Airfield and Highway Pavements.

[26] Kim M., Mohammad L.N., Elseifi M.A. (2015). Effects of various extrapolation techniques for abbreviated dynamic modulus test data on the MEPDG rutting predictions. J. Mar. Sci. Technol..

[27] Yildirim Y. (2007). Polymer modified asphalt binders. Constr. Build. Mater..

[28] (2015). Bitumen and Bitumen Binders-Determination of Needle Penetration.

[29] (2015). Bitumen and Bituminous Binders-Determination of the Softening Point-Ring and Ball Method.

[30] Arnevik R.E., Uthus N.S., Aurstad J., Aksnes J., Jørgensen T. (2019). Guidelines Asphalt 2019.

[31] (2011). Tests for Mechanical Physical Properties of Aggregates-Part 1: Determination of the Resistance to Wear (Micro-Deval).

[32] (2020). Tests for Mechanical and Physical Properties of Aggregates-Part 2: Methods for the Determination of Resistance to Fragmentation.

[33] Rowe G. (2009). Phase angle determination and interrelationships within bituminous materials. Advanced Testing and Characterization of Bituminous Materials, Two Volume Set.

[34] Fan B., Kazmer D.O. (2005). Low-temperature modeling of the time-temperature shift factor for polycarbonate. Adv. Polym. Technol..

[35] Tran N., Hall K. (2005). Evaluating the predictive equation in determining dynamic moduli of typical asphalt mixtures used in Arkansas. SAGE.

[36] Witczak M.W. (2002). Simple Performance Test for Superpave Mix Design.

[37] Veeraragavan A. (2011). Dynamic mechanical characterization of asphalt concrete mixes with modified asphalt binders. Mater. Sci. Eng. A.

[38] Dong F., Zhao W., Zhang Y., Wei J., Fan W., Yu Y., Wang Z. (2014). Influence of SBS and asphalt on SBS dispersion and the performance of modified asphalt. Constr. Build. Mater..

[39] Yu H., Shen S. (2012). Impact of aggregate packing on dynamic modulus of hot mix asphalt mixtures using three-dimensional discrete element method. Constr. Build. Mater..

